# HSYA alleviates secondary neuronal death through attenuating oxidative stress, inflammatory response, and neural apoptosis in SD rat spinal cord compression injury

**DOI:** 10.1186/s12974-017-0870-1

**Published:** 2017-05-03

**Authors:** Jun-peng Pei, Li-hong Fan, Kai Nan, Jia Li, Xiao-qian Dang, Kun-zheng Wang

**Affiliations:** 1 Department of Orthopaedics, the Second Affiliated Hospital of Xi’an Jiaotong University, No. 157 Xiwu Road, Xi’an, 710004 Shaanxi Province People’s Republic of China; 2 Department of Orthopaedics, the First Affiliated Hospital of Xi’an Jiaotong University, School of Medicine, Xi’an, 710061 China

**Keywords:** Apoptosis, Oxidative stress, Cytokines, Nuclear factor-kappa B (NF-κB), Spinal cord injury (SCI), Hydroxysafflor yellow A (HSYA)

## Abstract

**Background:**

Hydroxysafflor yellow A (HSYA) is a major active component of yellow pigment extracted from safflowers; this compound possesses potent neuroprotective effects both in vitro and in vivo. However, underlying mechanism of HSYA is not fully elucidated. The present study investigated the protective effects of HSYA in rat spinal cord compression injury model and related mechanisms involved.

**Methods:**

Sprague–Dawley rats were divided as Sham, Control, and HSYA groups (*n =* 30 per group). Spinal cord injury (SCI) model was induced by application of vascular clips (force of 50 g, 1 min) to the dura at T9–T10 level of vertebra. Injured animals were administered with either HSYA (8 mg/kg at 1 and 6 h after injury, then 14 mg/kg, for a total of 7 days at 24-h time intervals) or equal volume of saline by intraperitoneal injection.

**Results:**

From this experiment, we discovered that SCI in rats resulted in severe trauma, which is characterized by tissue damage, lipid peroxidation, neutrophil infiltration, inflammation mediator release, and neuronal apoptosis. However, HSYA treatment significantly reduced the following: (1) degree of tissue injury (histological score) and edema; (2) neutrophil infiltration (myeloperoxidase activity); (3) oxidative stress (superoxide dismutase, malondialdehyde, and nitric oxide); (4) pro-inflammatory cytokine expression (tumor necrosis factor-α, interleukin-6, inducible nitric oxide synthase, cyclooxygenase-2); (5) nuclear factor-κB activation; (6) apoptosis (terminal deoxynucleotidyl transferase dUTP nick end labeling staining and cysteine-aspartic protease-3 activity). Moreover, in a separate set of experiments, we clearly demonstrated that HSYA treatment significantly ameliorated recovery of limb function (as evaluated by Basso, Beattie, and Bresnahan behavioral recovery scores).

**Conclusions:**

Treatment with HSYA restrains development of oxidative stress, inflammation response, and apoptotic events associated with SCI of rats, demonstrating that HSYA is a potential neuroprotectant for human SCI therapy.

## Background

Spinal cord injury (SCI) is a medically, socioeconomically, and highly debilitating pathological condition, which often leads to catastrophic dysfunction and poses significant socioeconomic challenge worldwide [1]. Traumatic SCI is characterized not only by primary injury but also secondary neuronal death involving a large number of cellular, molecular, and biochemical cascades, which when controlled ineffectively, result in more extensive and sustained damages [2–4]. These pathological events can increase blood–spinal cord barrier (BSCB) permeability, tissue edema, peroxidation of lipid membranes, inflammatory cytokine release, and neural cell apoptosis [4, 5]. As SCI involves a multifactorial and interdependent complex pathophysiology [6, 7], an effective therapy should utilize either multiple agents or therapeutic compounds that can target multiple pathological mechanisms to prevent post-SCI neuronal death.

Numerous studies [2, 4, 8] pointed out that treatment between primary and secondary injury is extremely important because of the potential to either prevent or reduce final neurological deficits. To the authors’ best knowledge, though various pharmacological therapies were evaluated for treatment of SCI [9, 10], no therapy can effectively limit the evolution of secondary damage. Historically, after SCI, standard treatment regimen includes acute administration of high-dose corticosteroids [11]. However, such treatment results in neither conclusive nor consistent therapeutic effects [12, 13]; unexpected and dissatisfactory side effects can occur. Furthermore, molecular mechanisms of steroid action are still poorly known, disqualifying such therapy for further use in SCI therapeutic management. Therefore, new pharmacological therapies must be developed; such treatments should reduce evolution of secondary injury of SCI.

Hydroxysafflor yellow A (HSYA) is the main chemical component of yellow safflower pigments; it was demonstrated to have broad physiological and pharmacological functions, including anti-platelet agglomeration, anti-oxidation, anti-inflammation, and inhibition of apoptosis [14–18]. Studies showed that HSYA plays a protective role in animal models of pulmonary inflammatory injury [19, 20], cardiac failure [21, 22], and liver fibrosis [23, 24]. HSYA features extreme neuroprotection in animal models of acute and chronic central nervous system (CNS) injuries, including spinal cord ischemia, ischemia-reperfusion injury [17, 25, 26], and Parkinson’s disease [27]. Accumulated data suggest that HSYA can cure CNS lesions through mechanisms involving anti-inflammation and anti-oxidation. Thus far, no study attempted to describe the effects of HSYA and its detailed molecular mechanisms on SCI induced by clip compression.

Prompted by previous findings, the present study hypothesized that HSYA can effectively attenuate secondary damage of SCI by reducing oxidative stress, anti-inflammatory response, and neural apoptosis. Our results provide evidence that HSYA may constitute an effective therapeutic neuroprotectant for SCI.

## Methods

### Animals and materials

#### Animals

Ninety adult male Sprague–Dawley (SD) rats (weighing 270 to 330 g) were purchased from the Experimental Animals Center of Xi’an Jiaotong University. These rats were housed in a temperature-controlled environment (at room temperature of 22 ± 3 °C, relative humidity of 50 ± 15%, and 12-h light–dark cycle) supplied with standard rodent chow and water. All animal experimental procedures were conducted per the policies of our university, the Chinese authority, as well as the National Institute of Health Guide for the Care and Use of Laboratory Animals.

#### HSYA preparation

HSYA powder with high purity (>98% of the total weight) was purchased from Shandong Lvye Natural Medicine Research and Development Center (Shandong, China). HSYA solution was prepared and injected at a concentration of 1 mg/ml.

#### SCI model

Adequate analgesia was used throughout operation time with urethane (intraperitoneal injection (i.p.) 1.5 mg/kg body weight). The animals were placed in prone position. A dorsal 2-cm longitudinal incision was made over T9–T10 vertebrae, paraspinal muscles were dissected, and spinal process of T9–T10 were removed. Laminectomy of tenth vertebra was carried out to adequately expose the spinal cord. With 50 g closing force exerted, an aneurysm clip (Yasargil aneurism clip) was extradurally applied on spinal cords for 1 min. Skin was closed after clip removal. Average operation time was 30 min. Then, all rats were individually housed in a temperature-controlled room at 22 ± 3 °C for survival period of 48 h and 7 days. Water and food were provided to rats ad libitum. During this period, the animals’ bladders were manually voided twice a day until the rats regained normal bladder function.

#### Experimental design

All 90 adult SD rats were randomly allocated into the following three groups:

*HSYA group* (*N =* 30): rats were subjected to SCI and administered with HSYA (8 mg/kg, i.p. at 1 and 6 h after injury). Then, HSYA was further utilized once per day for 7 days (14 mg/kg, i.p. at 24 h interval) as one period of treatment.

*Control group* (*N =* 30): rats were subjected to SCI and injected with equal volume of saline.

*Sham group* (*N =* 30): rats were subjected to surgical procedures except for aneurysm clip application. Finally, all rats (*N =* 30 from each group) were sacrificed at 48 h (15 rats) and 7 days (15 rats) after SCI to evaluate schedule parameters.

To evaluate behavioral recovery effect of HSYA, in a separate set of experiments, another 18 animals for each group were observed from day 1 to 30 after SCI. Doses and time of HSYA treatment were selected based on previous in vivo studies [28, 29] and calculated dosage according to dose conversion formula between humans and rats.

### Functional scale

After SCI, behavioral test for motor function was performed once a day for 30 days. Functional recovery was evaluated according to the Basso, Beattie, and Bresnahan (BBB) scale [30], which is composed of 21 different criteria for movement of hindlimb (from no observable hindlimb movements to normal locomotion). This scale is based on precise observation of hindlimb movements, stepping, and coordination for 4 min in an open field by a trained observer, who is blinded to experimental conditions.

### Spinal cord edema formation

Spinal cord edema was evaluated by detecting water content of spinal cord. Spinal cord samples were weighed immediately (wet weight) when excised from lesions 48 h post-SCI. Then, injured spinal cords (*N =* 6 from each group) were dried for 48 h at 80 °C for determination of dry weight. Spinal cord edema was measured using the formula: spinal cord water content (WT) (%) = (wet weight − dry weight)/wet weight × 100%.

### Measurement of BSCB permeability

Permeability of BSCB was quantitatively evaluated using Evans blue dye extravasation per Uyama’s method [31] but with certain modifications. In brief, 48 h after SCI, rats were anesthetized, and 4 mL/kg of 2.5% Evans blue dye (Sigma) solution in saline was administered through the femoral vein. One hour later, all animals were killed by intra-cardiac perfusion with 250 mL of saline. The T10 spinal cord segment was removed and placed in 50% trichloroacetic acid (TBA) solution. After homogenization and centrifugation, optical density of extracted dye was measured at 620 nm with a spectrophotometer. In spinal cord tissues, quantitative calculation of dye content was determined as micrograms per gram (μg/g) of tissue from a standard curve plotted using known amounts of dye.

### Spinal cord biochemical analysis

#### Preparation of spinal cord homogenate

As soon as the rats were sacrificed, their spinal cords (*N =* 6 from each group) were quickly removed to collect lesion segments for all biochemical assays. Tissue samples were washed by frozen saline and immediately prepared as homogenates (1:10) then centrifuged (14,000 rpm, 4 °C, 15 min); supernatant layer was derived and immediately frozen in liquid nitrogen and stored at −70 °C until further processing. Protein assays were conducted using protein assay kits (Nanjing Jiancheng Bioengineering Institute, Nanjing, China).

#### Tissue malondialdehyde (MDA) analysis

In spinal cord samples, lipid peroxidation was determined as MDA concentration. After injury, MDA levels in damaged spinal cord were measured based on reaction with TBA using MDA assay kits (Nanjing Jiancheng Bioengineering Institute, Nanjing, China) per manufacturer’s instructions. Using N-methyl-2-phenylindole as substrate, intracellular MDA concentration was calculated by measuring maximal absorbance at 532 nm on a spectrophotometer. MDA concentrations were expressed as nanomoles per milligram of spinal cord protein (nmol/mg prot).

#### Myeloperoxidase (MPO) measurement

MPO activity is an indicator of polymorphonuclear leukocyte (PMNL) accumulation; it was determined in spinal cord homogenate with rat MPO assay kits (Nanjing Jiancheng Bioengineering Institute, Nanjing, China) per manufacturer’s instructions. Samples were measured on a spectrophotometer at 460 nm absorbance. MPO activity was defined as quantity of enzyme degrading 1 μmol of peroxide per min at 37 °C and was expressed as units per milligram of spinal cord protein (U/mg prot).

#### Nitric oxide (NO) determination

As NO rapidly converts into nitrate and nitrite, and its measurement is very difficult in biological specimens, total NO production in samples was measured based on Griess reaction [32]. Reduction of nitrate to nitrite was accomplished by catalytic reaction using cadmium. Total nitrite (nitrate + nitrite) was determined by diazotization of sulfanilamide and coupling to naphthylethylene diamine. Absorbance of this complex was measured at 540 nm. Results were expressed as nanomoles per milligram of spinal cord protein (nmol/mg prot) in spinal cord tissue.

#### Superoxide dismutase (SOD) assay

Total SOD activity in spinal cord homogenate was measured with xanthine oxidase assay kits (Nanjing Jiancheng, Bioengineering Institute, Nanjing, China) per manufacturer’s instructions. The principle of this method is based on inhibition of nitroblue tetrazolium (NBT) reduction by xanthine–xanthine oxidase system as superoxide generator. One unit of SOD was defined as the enzyme amount causing 50% inhibition in NBT reduction. SOD activity was expressed as units per milligram of spinal cord protein (U/mg prot).

### Enzyme-linked immunosorbent assay (ELISA) measurement of tumor necrosis factor (TNF)-α and interleukin (IL)-6

To test whether spinal cord damage was associated with pro-inflammatory cytokine formation, we measured TNF-α and IL-6 levels in perilesional spinal cord tissue by ELISA (R&D Systems, Minneapolis, MN). Spinal cord tissue lesion samples were prepared as previously described. ELISAs of spinal cord tissues were performed per manufacturer’s instructions. All assays were carried out in duplicate using recommended buffers, diluents, and substrates. Cytokine concentrations in tissues were measured as picogram per milligram of wet tissue.

### Electrophoretic mobility shift assay (EMSA) and Western blot analysis

Nuclear extracts from spinal cord tissues were isolated and quantified as previously described [33, 34]. EMSA was performed using a commercial kit (Light Shift Chemiluminescent EMSA Kit; Pierce; Rockford; USA) following the methods in our laboratory. Double-stranded oligonucleotides containing nuclear transcription factor-κB (NF-κB) recognition sequence (5′-AGTTGAGGGGACTTTCCCAGGC-3′) were end-labeled with γ^32^-P adenosine triphosphate and T4 polynucleotide kinase. Nuclear extract (10 μg) was incubated for 20 min with gel-shift-binding buffer and 2 μL of radio labeled probe. Nuclear protein with 32P-labeled oligonucleotide complex was separated from free 32P-labeled oligonucleotide by electrophoresis using 5% native polyacrylamide gel in a running buffer. The gels were then vacuum-dried for autoradiography. Subsequently, relative bands were quantified by densitometric scanning. For competition assays, a 200-fold molar excess of unlabeled NF-κB oligonucleotide was added to binding reaction.

To confirm NF-κB expression using different testing methods, we performed a Western blot; activation of cysteine-aspartic protease(caspase)-3 was also tested. Procedures were all performed per manufacturer’s instructions (BCA Protein Assay Kit). Briefly, frozen homogenate tissue samples were thawed and incubated for 30 min at 4 °C and centrifuged at 4 °C for 10 min at 14,000 rpm. Supernatants were then collected. Protein lysate buffer was added to 20 mg of frozen tissues (with 2.0 μl protein lysate per 1.0 mg tissue). Each sample containing 20 μg total protein was loaded onto a 12% separating gel and transferred to membranes. After washing once with Tris buffered saline with Tween 20 (TBST) for 5 min and blocking in 5% skim milk in TBST for 4 h at room temperature, the membranes were incubated overnight with primary antibodies, anti-NF-κB (1:100, Santa Cruz, CA), and anti-β-actin (1:2000) at 4 °C. After washing, membranes were incubated with horseradish-peroxidase (HRP)-labeled goat anti-mouse (1:10,000) secondary antibody (Jackson) for 4 h at room temperature. Color was developed using enhanced chemiluminescence, and images were analyzed with LabWorks gradation image analysis software. Each experiment was repeated thrice.

### Light microscopy

#### Hematoxylin/eosin (HE) staining

Spinal cord biopsies (*N =* 6 from each group) were taken at 48 h and 7 days after trauma. On each side of lesion sites, 1 cm tissue segments were fixed for 24 h in paraformaldehyde solution (4% in 0.1 M phosphate-buffered saline (PBS)) at room temperature, dehydrated by graded ethanol, embedded in Paraplast, and cut into 5-μm-thick sections. Tissue sections were deparaffinized with xylene, stained with HE, and finally studied using light microscopy. An experienced histopathologist evaluated each spinal cord segments in a blinded fashion. Histopathological changes in gray matter were studied and scored using a 6-point scale: 0, no lesion observed; 1, gray matter contained 1–5 eosinophilic neurons; 2, gray matter contained 5–10 eosinophilic neurons; 3, gray matter contained more than 10 eosinophilic neurons; 4, small infarction (less than one third of the gray matter area); 5, moderate infarction (one third to one half of the gray matter area); 6, large infarction (more than half of the gray matter area). From each spinal cord, scores of histological sections were averaged to obtain a final score for individual animals.

#### Nissl staining

Selected 5-μm-thick sections were dewaxed and rehydrated gradually, immersed in 0.5% cresylviolet for 2 min, rinsed with double distilled water, dehydrated in ethanol solutions with increasing concentrations, and cleared in xylene. Next, the sections were mounted with Permount cover slip and observed under a light microscope. Normal neurons included those containing Nissl substance in cytoplasm, loose chromatin, and prominent nucleoli; damaged ones comprised those without Nissl substance, cavitation around nucleus, and pyknotic homogenous nuclei.

#### Immunohistochemical staining

For immunocytochemistry, endogenous peroxidase was quenched with 0.3% (*v*/*v*) hydrogen peroxide in 60% (*v*/*v*) methanol for 30 min after deparaffinization [35]. Then, to block non-specific adsorption, all specimens were incubated in 2% (*v*/*v*) normal goat serum. Endogenous biotin or avidin-binding sites were blocked by sequential incubation for 15 min with biotin and avidin. Sections were incubated overnight with anti-cyclooxygenase (COX)-2 antibody (Santa Cruz Biotechnology, 1:500 in PBS, *v*/*v*) and anti-inducible-nitric-oxide synthase (iNOS) antibody (Santa Cruz Biotechnology, 1:500 in PBS, *v*/*v*). Sections were then washed with PBS and incubated with secondary antibodies. Specific labeling was detected with biotin-conjugated goat anti-rabbit IgG and avidin-biotin peroxidase complex (Vector Laboratories, DBA). In all controls, reaction to substrate was absent when primary antibody was omitted, or when primary antibody was replaced by a nonimmune control antibody. For quantitative analysis, original immunohistochemical photographs (*n =* 5 photos from each sample collected from all rats in each experimental group) were assessed by densitometer using MacBiophotonics Image J 1.41a software on a personal computer.

#### Terminal-deoxynucleoitidyl transferase (TdT) mediated nick end labeling (TUNEL) assay

TUNEL assays were performed using a TUNEL detection kit according to manufacturer’s instruction (Apotag HRP kit; DBA) and guidelines of TUNEL assay [36]. After fixation, tissue sections were incubated with 15 μg/ml proteinase K for 15 min at room temperature and washed with PBS. Endogenous peroxidase was inactivated by 3% H_2_O_2_ for 5 min at room temperature and then washed with PBS. Sections were incubated in TdT buffer containing deoxynucleotidyl transferase and biotinylated deoxyuridine triphosphate in TdT buffer, incubated in humid atmosphere at 37 °C for 90 min, and then washed with PBS. Tissue sections were also incubated at room temperature for 30 min with anti-HRPY-conjugated antibody, and signals were visualized with diaminobenzidine. In spinal cord, TUNEL-positive cells from penumbra of lesions were quantified by counting positively stained cells. An average of five sections (100 μm apart) from each animal (*n =* 6) were analyzed, and data were expressed as number of cells per section.

### Statistical evaluation

All data were reported as mean ± standard deviation. All statistical analyses were conducted using statistical analysis software (SPSS, Version 18.0). In experiments involving histology or immunohistochemistry, all figures shown are representative of at least three experiments performed on different experimental days. Results were analyzed by one-way ANOVA followed by Bonferroni post hoc test for multiple comparisons. BBB scale data were analyzed by Mann–Whitney test. *P* values of less than 0.05 were considered statistically significant.

## Results

### Functional recovery

**Fig. 1:**
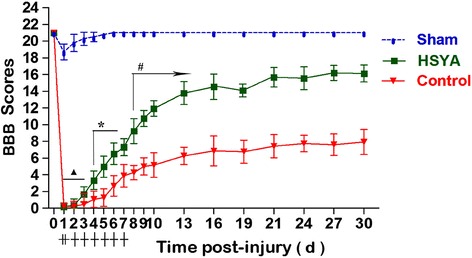
Neurological function measured by BBB locomotion scores from 0 to 30 days after SCI. In general, functional recovery gradually improved both in HSYA and control groups from day 2 after SCI. During the whole experimental period (except for the first 2 days), HSYA-treated group showed significantly better function in comparison with the control group. In the sham group, neurological functions of rats were slightly damaged but were all restored quickly within 7 days. (*n =* 18 in each group, ▲*P >* 0.05; *P < 0.05; #*P <* 0.01, HSYA vs. Control group)

All rats used in this study started with a BBB locomotion score. Motor function of rats was only slightly impaired in sham group and was recovered quickly within a week. Rats subjected to SCI showed complete bilateral hindlimb paralysis. In general, in comparison with the control group, behavior of HSYA treatment group improved more rapidly from day 3 until day 13 (13.77 ± 1.35 vs. 6.26 ± 1.04) post-trauma, revealing their capacity to consistently support weight on plantar steps and consistent forelimb–hindlimb coordination. Improvement was maintained until terminal point day 30 (Fig. 1). Although control group animals progressively regained some motor functions after the fifth day, their BBB scores were still significantly lower in comparison with that of HSYA-treated group (1.27 ± 1.06 vs.4.96 ± 1.27). These data clearly revealed that HSYA treatment significantly improved disturbances in hindlimb motor after SCI.

### HSYA decreases tissue edema and BSCB permeability

**Fig. 2:**
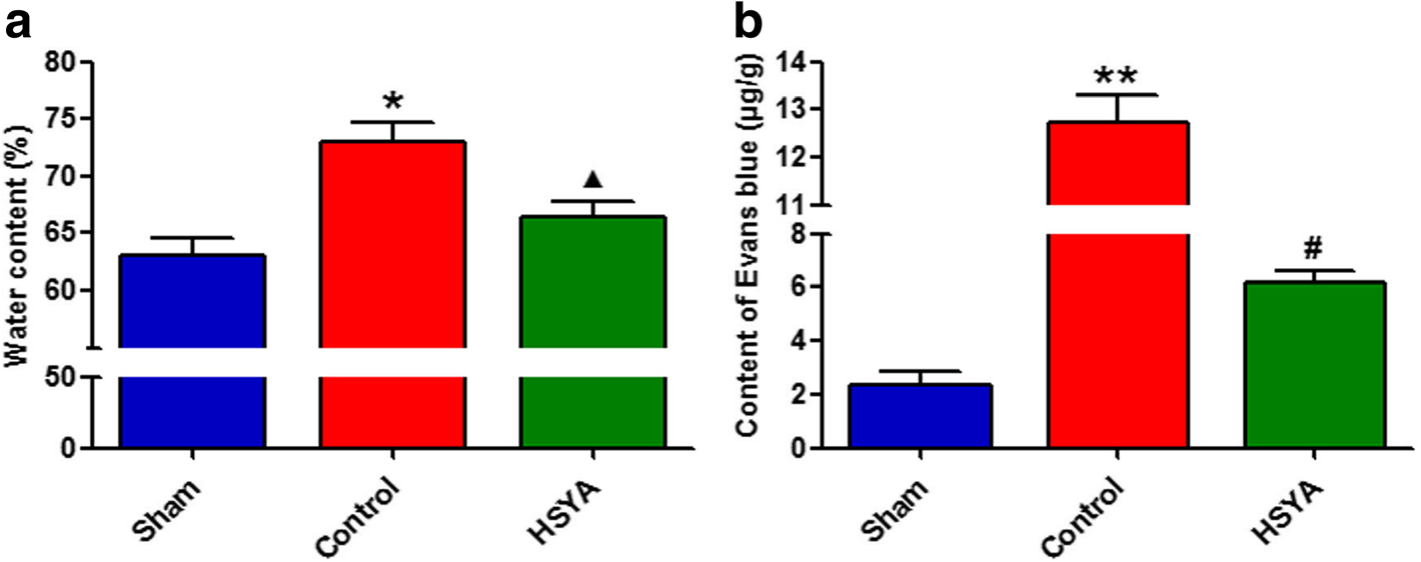
Changes of water content (**a**) and BSCB permeability (**b**) in the injured spinal cord at 48 h after SCI. Compared with the sham group, the trauma caused an increased amount of water content and Evans *blue* extravasation at 48 h after SCI. Treatment with HSYA significantly attenuated both amount of water content and Evans blue leakage as compared with the vehicle- treated control group. (*n =* 6 in each group, *P < 0.05 vs. sham group; **P < 0.01 vs. sham group; ^▲^ *P <* 0.05 vs. sham group; *P <* 0.01 vs. control group; #*P <* 0.01 vs. control & sham group)

After 48 h, SCI spinal cord edema formation significantly increased in comparison with the sham group. Interestingly, administration of HSYA significantly reduced water content of spinal cord, but statistical difference was still noted between sham and HSYA-treated animals (*P <* 0.05, Fig. 2a).

To determine whether HSYA attenuates SCI-induced increased permeability in BSCB, Evans blue content was measured at 48 h post-SCI; Evans blue is a marker of blood–CNS barrier disruption. As shown in Fig. 2b and as indicated by Evans blue extravasation, 50 g closing force with aneurysm clip profoundly increased focal trauma to the spinal cord in comparison with the sham group 48 h after injury (*P <* 0.01). Simultaneously, in HSYA-treated group, Evans blue in spinal cord tissue significantly decreased in comparison with the control group (*P <* 0.01), demonstrating that HSYA treatment alleviated increase in BSCB permeability against SCI insult.

### Effects of HSYA on oxidant stress and inflammatory cytokines after SCI

**Fig. 3:**
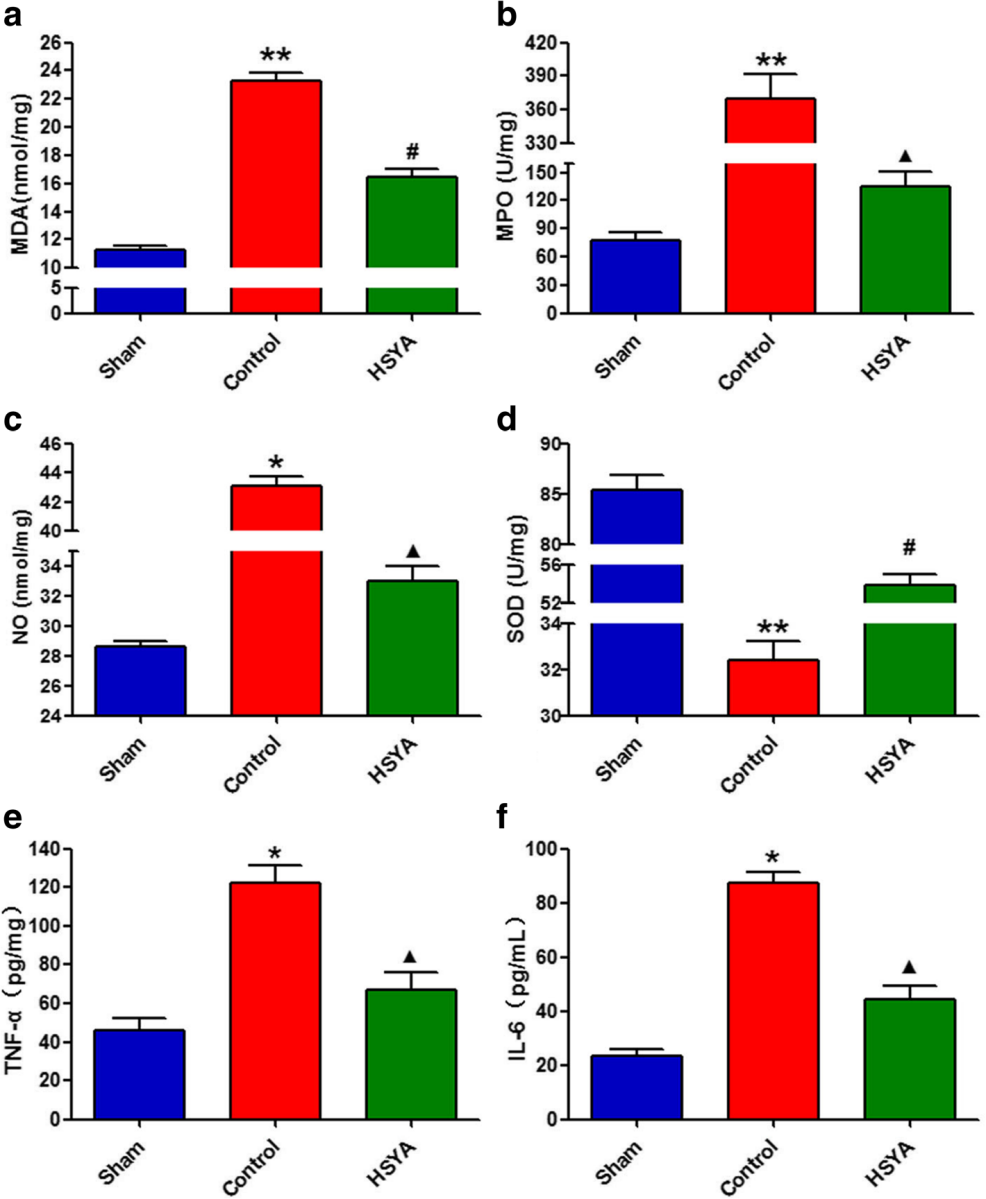
Quantification of oxidant stress markers and inflammatory cytokines in spinal cord. **a–c** Consistent oxidant stress marker parameter results of MDA, MPO, and NO in the control group revealed significantly increase as compared with the sham group 48 h after the lesion. Unanimous as well, these increases were significantly attenuated by HSYA treatment. **d** Compared with the sham group, the trauma reduced the amount of SOD at 48 h after SCI; however, treatment with HSYA significantly recuperated the amount of SOD. **e, f** Inflammatory cytokines in spinal cord tissue were detected by ELISA. The levels of TNF-α and IL-6 were significantly increased at 48 h after SCI. On the contrary, HSYA treatment attenuated the SCI-induced increases of these cytokines. (*n =* 6 *P < 0.05 vs. sham group; **P < 0.01 vs. sham group; ^▲^ *P <* 0.05 vs. sham group; *P <* 0.01 vs. control group; #*P <* 0.01 vs. control & sham group)

Figure 3a–d presents statistical evaluations of MDA and NO levels and enzymatic activity (SOD and MPO) detected by biochemical analysis of spinal cord tissues. In the control group, SCI significantly increased levels of MDA, MPO, and NO in spinal cord tissue in comparison with the sham group. Meanwhile, HSYA treatment effectively prevented these increases. On the contrary, SOD activity in control group significantly decreased in comparison with that of sham group and was well-protected and preserved by HSYA treatment.

Results of ELISA (spinal cord tissues samples collected from lesion sites 48 h after SCI) demonstrated substantial increase in TNF-α and IL-6 productions. However, HSYA treatment significantly reduced levels of TNF-α and IL-6 in spinal cord in comparison with that in control group. Results suggest that SCI can induce production of pro-inflammatory cytokines in spinal cord, and this effect can be significantly alleviated by HSYA treatment.

### HSYA inhibits NF-κB activation

**Fig. 4:**
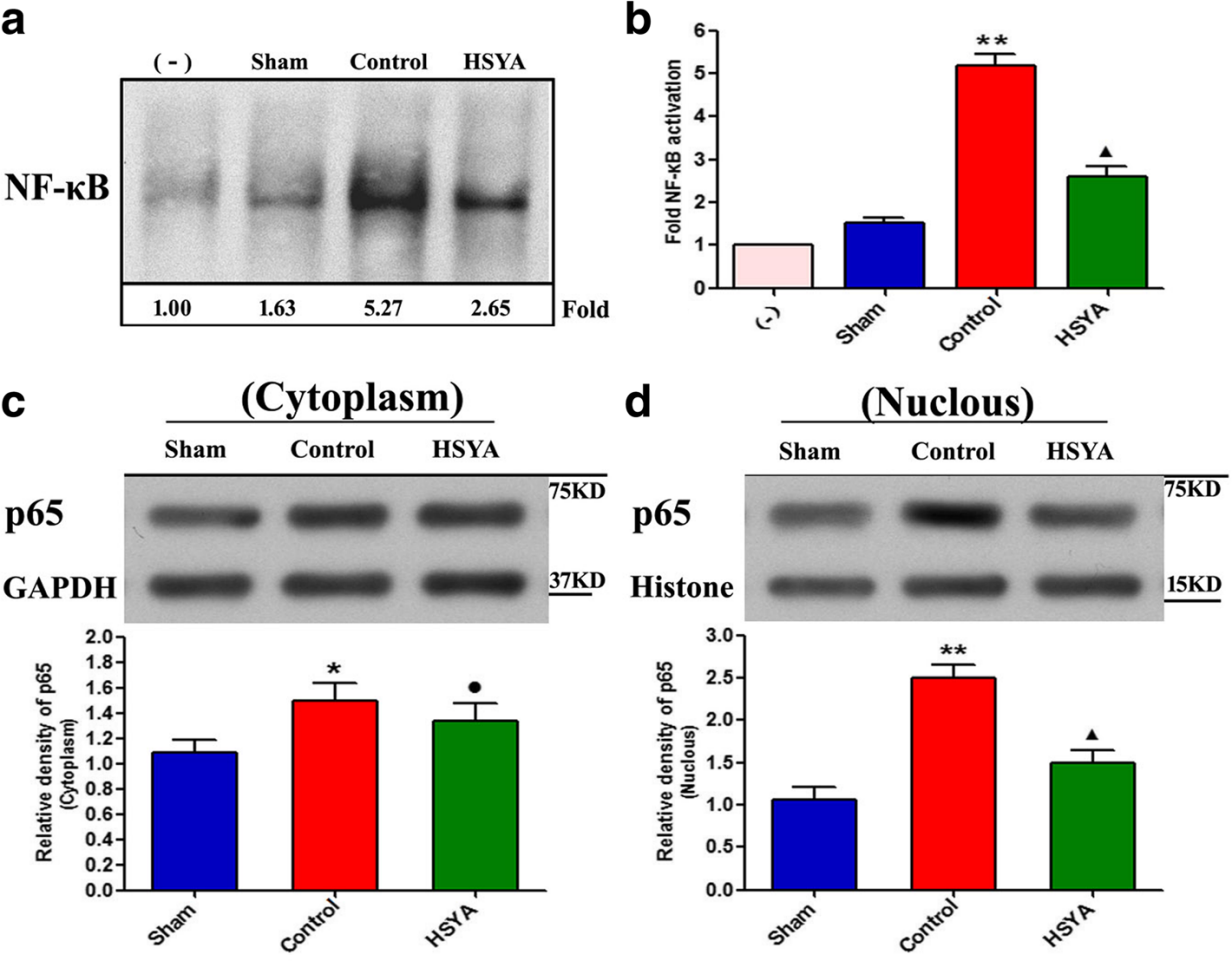
Effect of HSYA on NF-κB activation in rat spinal cord. **a, b** Representative result from EMSA shows DNA-binding activity of NF-κB was at very low levels in the sham group but significantly increased in the control group, but significantly decreased after HSYA treatment. **c, d** The activity of NF-κB by Western blot, results of NF-κB nucleus importation was unanimous as that of EMSA, but no significant differences were observed on NF-κB in cytoplasm. This figure is representative of three experiments performed on different experimental days. (*P < 0.05 vs. sham group; ●*P >* 0.05 vs. control group; *P <* 0.05 vs. sham group; **P < 0.01 vs. sham group; ^▲^ *P <* 0.05 vs. sham group; *P <* 0.01 vs. control group)

To investigate whether changes in inflammatory cytokines in spinal cord are associated with attenuation of NF-κB activation, NF-κB/DNA binding was analyzed by EMSA. The top section of Fig. 4a, b shows representative EMSA image and relative quantitative data obtained on NF-κB activation. DNA-binding activity of NF-κB was present at very low levels in the sham group but significantly increased in the control group. However, NF-κB activation markedly decreased in HSYA-treated group in comparison with the control group (*P <* 0.05). Upon further detection of activity of NF-κB by Western blot (c and d), results of NF-κB nucleus importation was unanimous as that of EMSA, but no significant differences were observed on NF-κB in cytoplasm, revealing imparity distribution after SCI and probable blocking effect of NF-κB translocation.

### HSYA attenuates severity of SCI

**Fig. 5:**
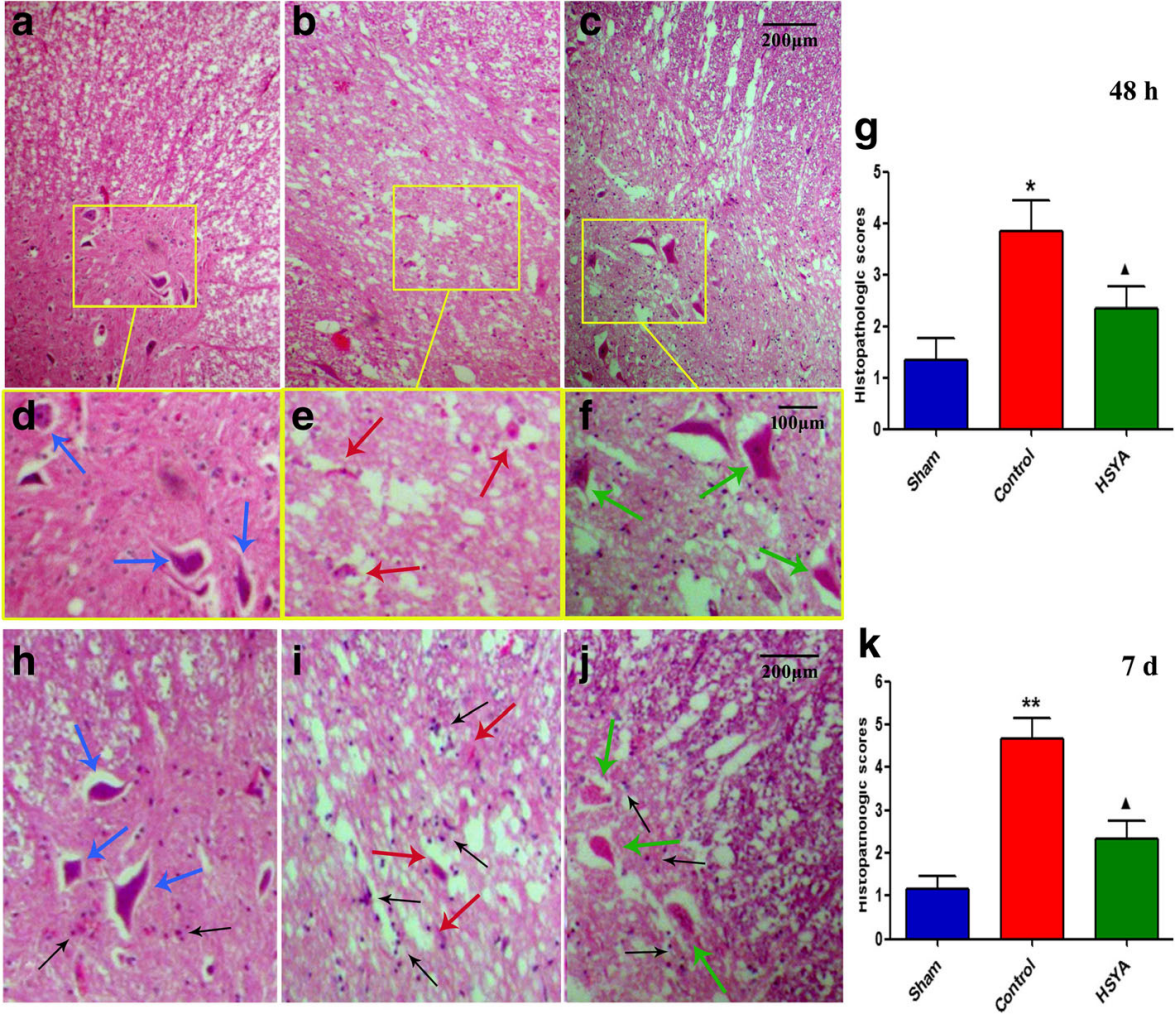
HE staining of the spinal cord segments at the perilesional area 48 h and 7 days after SCI. **a–f** Segments at the perilesional area 48 h after SCI. **a, d** Normal appearance of neurons in sham-operated rat; (**b, e**) Spinal cord section of a rat 48 h after SCI with paraplegia, showing necrotic tissue with severe neuronal damage was evidenced by eosinophilic neuronal degeneration. **c, f** Near to normal appearance neurons of the anterior horn of a T9–10 spinal cord section from a HSYA-treated rat. Though more white sections remained after much more water content were dehydrated (compared to **a**), both spinal cord tissue integration and most motor neurons were preserved against SCI insult. **h–j** HE staining 7 days after SCI. In the control group, typical necrosis exhibited and characterized as broad edema, reactive gliosis, and neural apoptosis with condensed nuclei. When compared with the sham group, a significant protection from SCI-associated damage was observed in the spinal cord tissue collected from HSYA-treated rats, showed normal-like morphology with clear boundary around neural and glial. Compared with 48 h, much more neuroglia cells characterized its morphology at 7 days after SCI (*black arrow*), SCI much increased the number of glia cells mainly around neuro-cells in the control group compared with the sham group, and HSYA treatment effectively prevented the increase. The histopathologic score was made by the presence of edema as well as alteration of the gray matter 48 h and 7 days after SCI from an independent observer (**j, k**). (Original magnification 200; *n =* 6 per group. *P < 0.05 vs. sham group; **P < 0.01 vs. sham group; ^▲^ *P <* 0.05 vs. sham group and control group)

At perilesional area, damage severity was evaluated at 48 h and 7 days after SCI; this evaluation assessed the presence of edema, alteration of gray matter, and infiltration of leukocytes. As shown in Fig. 5, at both time points, significant alteration of morphology was observed in spinal cord tissues of SCI rats in comparison with the sham group, and HSYA-treated rats experienced protection against alteration of neuron’s morphology. Seven days after SCI, results of HE staining were used for further therapeutic effect assessment; observations were similar with HE staining after 48 h. Notably, when histopathological changes in tissue sections were scored, rats in the control group incurred relatively higher scores in comparison with the sham group and HSYA-treated group; HSYA treatment significantly reduced histopathological scores associated with SCI.

### Effects of HSYA on expression of COX-2 and iNOS

**Fig. 6:**
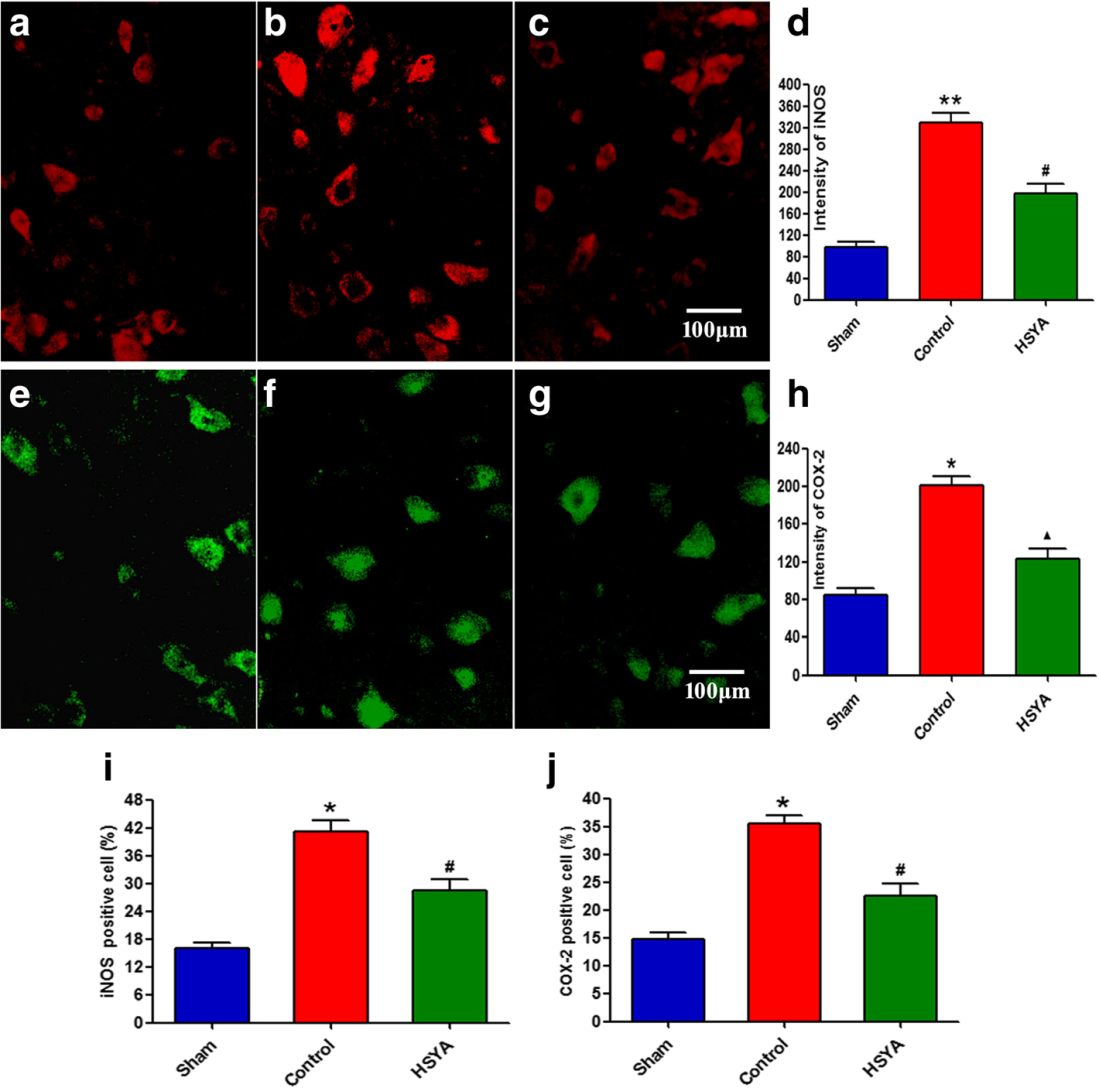
The immunohistochemical staining of iNOS (**a** to **c**) and COX-2 (**e** to **g**) 48 h after SCI. The quantitative analysis of iNOS and COX-2 from spinal cord tissues in each experimental group has been assessed; bar graphs represent the mean optical density of iNOS (**d**) and COX-2 (**h**), respectively. iNOS and COX-2-positive apoptotic cells (**i** and **j**, respectively) in spinal cord lesions were also quantified by counting the total number of positive nuclei through entire cross sections by an independent observer. Quantitative analysis was based on at least five fields per section of five rats, separately. (*P < 0.05 vs. sham group; **P < 0.01 vs. sham group; ^▲^ *P <* 0.05 vs. sham group; *P <* 0.01 vs. control group; #*P <* 0.01 vs. control and sham group)

As shown in Fig. 6, 48 h after SCI, lesion sections of T10 spinal cord were obtained for immunohistological staining to detect iNOS and COX-2 expressions. iNOS was less and weakly stained in the spinal cord obtained from the sham group rats (a). Spinal cord sections obtained from control animals exhibited significantly positive staining for iNOS (b). Inducible COX-2 staining was scarcely observed in some neurons among the gray matter of sham animals (e). Substantial increase in iNOS reactivity was noted in spinal cord tissues collected from SCI rats 48 h after SCI (f). Levels of iNOS and COX-2 were significantly attenuated by HSYA treatment (c and g).

For quantitative analysis, immunohistochemical photographs were assessed by densitometer using MacBiophotonics Image J 1.41a software on a personal computer. The results (precise optical density data presented as d and h) also demonstrated significantly higher intensity of iNOS and COX-2 in SCI rats in comparison with sham-operated animals. Meanwhile, HSYA treatment significantly reduced accumulation of iNOS and COX-2 associated with SCI. In spinal cord lesions, iNOS and COX-2 positive apoptotic cells were quantified; result is consistent with that of optical density.

### HSYA attenuates apoptosis and caspase-3 activity

**Fig. 7:**
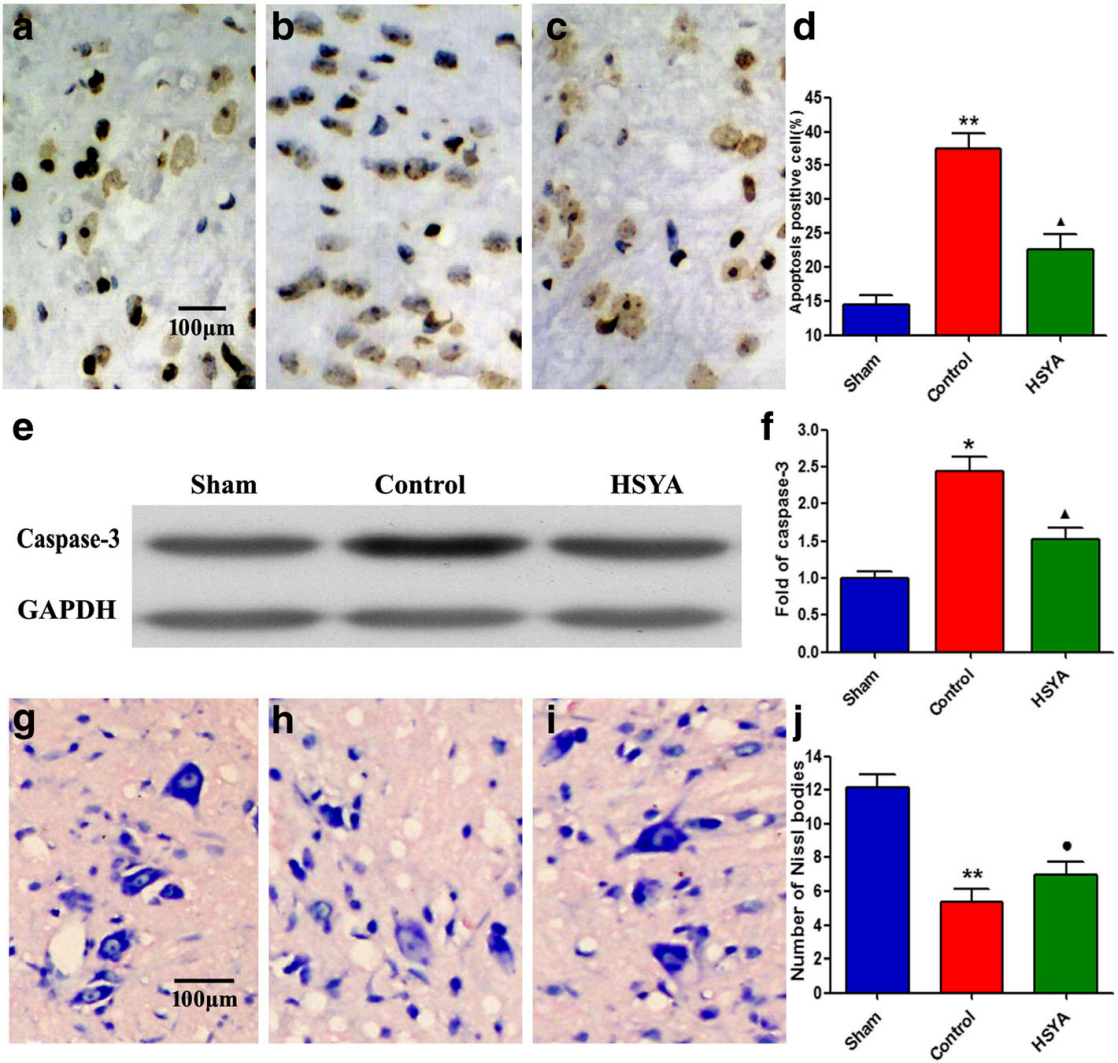
HSYA treatment reduced the motor neuron apoptosis of spinal cord at 48 h after SCI. The motor neuron apoptosis was detected by TUNEL staining and Western blot of caspase-3 activity. Obviously, much more karyopyknosis, nuclear fragmentation could be observed in the control group (**b**) as compared with the sham group (**a**). Meanwhile, HSYA treatment (**c**) obviously reduced the quantity of TUNEL-positive nuclei, but still more than that of sham group. The nuclear shape of motor neurons was well preserved in the HSYA group. **e, f** The Western blotting results showed that the caspase-3 activity was significantly increased in spinal cord injury animals, which was significantly reduced by HSYA treatment. **g-j** Nissl staining: In the sham group (**g**), a large amount of granule-like and densely stained toluidine blue were observed in the neurons’ cytoplasm. However, Nissl bodies significantly decreased in the control group (**h**) neurons. In the HSYA group (**i**), the number of Nissl bodies preserved compared with that of the control group, whose morphology more resembles that of the sham group. (*P < 0.05 vs. sham group; **P < 0.01 vs. sham group; ^▲^ *P <* 0.05 vs. sham group and control group; ●*P <* 0.05 vs. control group; *P >* 0.05 vs. sham)

To test whether neuroprotective effects were associated with neural apoptosis inhibition, we measured TUNEL-like staining in perilesional spinal cord tissues. As shown in Fig. 7, fewer apoptotic cells were detected in spinal cords of sham group (a). However, 48 h post-SCI, many TUNEL-positive neuronal cells in control group demonstrated marked appearance of dark brown apoptotic cells and intercellular apoptotic fragments, which are indicative of neuronal apoptosis (b). Almost no differences were found in HSYA group (c) in comparison with the sham group. Western blot results showed that caspase-3 activity (e and f) significantly increased in SCI animals; this phenomenon was significantly reduced by HSYA treatment. These results suggest that spinal cord compression injury can increase motor neuron apoptosis of spinal cord, and this condition can be significantly attenuated by HSYA treatment.

After dewaxing and rehydration of lesion tissues of SCI rats, Nissl staining (g to j) was also conducted to further confirm pathological damages. Results showed well-maintained nerve cells in the gray matter of the spinal cord in the sham group, whereas the control group presented large population of damaged and swollen nerve cells with vacuolated cytoplasm and disintegrated nucleus. Interestingly, HSYA treatment group exhibited markedly alleviated damages.

## Discussion

In the present study, we demonstrated that HSYA treatment (8 mg/kg injection for 1 and 6 h after SCI and 14 mg/kg once daily until day 7) significantly decreased damage severity and effectively attenuated neurological dysfunction after SCI (edema, BSCB permeability, and histological score). We also observed that beneficial effects of HSYA treatment against SCI were associated with decreased levels of oxidative products (MDA, NO, and MPO), reduction of pro-inflammatory cytokines (TNF-α, IL-6, iNOS, and COX-2) and NF-κB activation, and increased activities of antioxidant enzymes (SOD). HSYA significantly ameliorated caspase-3 activity and neural apoptosis. These findings suggest that HSYA is useful in treating SCI by reducing oxidative stress, inflammatory response, and caspase-related apoptosis.

Combination of acute impact followed by persisting compression is the most common mechanism of SCI in humans [6]. To mimic mechanical events and to evaluate protective effects of HSYA on SCI, we conducted an experimental study on rats using validated aneurysm compression clip model [37]. Clip blades simultaneously exert force on the spinal cord from ventral and dorsal surfaces, simulating mechanisms in human SCI; such mechanisms include fracture-dislocation and burst-compression fractures. SCI leads to pathological cascades that irreversibly exacerbate primary injury, trigger secondary injury, and result in permanent functional deficits [38, 39]. This synergistic pathophysiology involves many mechanisms, including inflammation, glutamate excitotoxicity, apoptosis, edema, and oxidative stress [40]. When secondary injury is increased because of delayed treatment, injury cascade is possibly more extensive and autodestructive [39, 41]. Early therapeutic treatment with drugs can be simultaneously more effective; such drugs should target more than one mechanism or target multiple pathways.

In traditional oriental medicine, flowers of safflower plant, *Carthamus tinctorius* L., are extensively used for treatment of cerebrovascular and cardiovascular diseases [42]. Extracts of *C. tinctorius* L. contain yellow and red pigments HSYA, safflor yellow B, safflomin A, safflomin C, and other compounds. HSYA is the major active chemical component of yellow color pigments extracted from safflower; it was demonstrated to have potent antioxidative and neuroprotective effects in vitro and in vivo [29, 43]. Recent studies revealed that in rats, HSYA can alleviate lipopolysaccharide-induced lung injury [19] and ischemia-reperfusion-induced brain injury by inhibiting inflammatory responses [25]. Studies also reported that HSYA protects human umbilical vein endothelial cells against hypoxic injury by inhibiting cell apoptosis [44, 45]. Our previous in vitro [46] study demonstrated that HSYA can block oxygen and glucose deprivation followed by reperfusion (OGD-R) induced pc-12 apoptosis through suppression of intracellular oxidative stress and mitochondria-dependent caspase cascade. In the present in vivo study, we discovered that HSYA treatment significantly improved hindlimb motor disturbances after SCI. In addition, histopathological results showed that HSYA protected spinal cord damage caused by clip compression. Moreover, HSYA not only preserved water content ratio of spinal cord and reduced permeability of BSCB but inhibited spinal cord neutrophil recruitment as well. All the above results showed protective effects of HSYA treatment against aneurysm-clip-induced spinal cord compression injury.

Although the definitive pathophysiology regarding SCI still remains obscure, oxidative stress mediators are suggested to play a central role; these mediators include reactive oxygen species (ROS), PMNL, and NO [12, 47–49]. Free-radical-oxygen-induced lipid peroxidation was suggested to be one of the most important factors precipitating posttraumatic degeneration in spinal cord [50]. Spinal cord tissue is particularly vulnerable to free radical oxidation following hypoxic or traumatic insults because of its high lipid content and poor iron-binding capacity [51]. In the present study, we first investigated HSYA’s influence on production of ROS, which contributes to prognosis of SCI; oxidative stress was monitored by measuring MDA and SOD, which are the ultimate products of unsaturated lipid peroxidation and provide information on lipid peroxidation and antioxidant defense of organisms [52]. We observed that 48 h after SCI, MDA level significantly increased, and SOD activity markedly decreased; these parameters were evidently improved by HSYA treatment. MPO is an enzyme located mainly in primary granules of neutrophils; MPO levels in tissues may suggest leukocyte infiltration into spinal cord tissue and generate reactive oxygen and nitrogen species and proteases [53, 54]. Studies suggested that overproduction of NO generated by iNOS not only aggravates oxidative damage and DNA modification but also leads to microvascular dysfunction [55]. Our present study demonstrated that after 48 h, MPO and nitrotyrosine significantly increased in the control group; both were significantly reduced by HSYA treatment. Therefore, our data strongly suggest that as an antioxidant, HSYA interferes in scavenging of free radicals; this action is possibly its basic protection mechanism.

Previous studies [56–58] showed potential anti-inflammatory effect of HSYA. Pro-inflammatory cytokines play important roles in initiation and amplification of inflammatory responses in secondary damage after SCI; pro-inflammatory cytokines include TNF-α, IL-6, and inflammatory mediators, such as iNOS and COX-2. Inhibiting overproduction of these pro-inflammatory cytokines and mediators decreased spinal cord damage in various traumatic and ischemic SCIs [59, 60]. Thus, blocking the effects of pro-inflammatory mediators offers an attractive therapeutic strategy. In this experiment, we investigated the possibility that HSYA acts as anti-inflammatory agent on SCI-induced inflammatory response. Our findings provide evidence that although concentrations of TNF-α and IL-6 and expression of COX-2 and iNOS in spinal cord significantly increased after SCI, they were markedly reduced after HSYA treatment. NF-κB plays a central role in regulation of numerous genes responsible for generation of mediators or proteins in CNS inflammation response. Activation of NF-κB results in expression of various pro-inflammatory molecules, such as TNF-α, IL-1, IL-6, and iNOS, causing neuronal death [61]. In previous studies, scientists proved that hydrogen can inhibit activation of NF-κB both in vitro and in vivo [62, 63]. Therefore, in the present study, we evaluated the effects of HSYA on NF-κB activation in vivo to further obtain dependable evidence. EMSA was conducted to determine transcription activity of NF-κB, further revealing significantly increased activation of this factor after SCI; this result was strongly reversed by HSYA treatment. Considering all the above results, our findings suggest neuroprotective effect of HSYA on an injured spinal cord; such effect is partly due to inhibition of inflammation. Thus, HSYA may be used as a potential therapeutic agent against inflammation in other CNS diseases.

Apoptosis is programmed cell death, which is characterized by specific ultrastructural changes, including cell shrinkage, nuclear condensation, and DNA fragmentation [64]. Excessive ROS and inflammatory cytokine can result in lipid peroxidation, inactivation of protein, and DNA fragmentation, leading to apoptosis or necrosis depending on severity of oxidative stress [65]. Apoptosis was demonstrated to be an important mode of neuronal death after SCI and plays a crucial role in paraplegia [66]. To determine whether inhibition of apoptosis was involved in protective effects of HSYA on SCI, we examined spinal cord neural cell apoptosis by TUNEL staining. Our results showed that in HSYA group, SCI-induced TUNEL-positive staining significantly decreased in comparison with those in the control group. At a molecular level, apoptosis is activated by caspase cascades, including those involving caspase-12 and caspase-3. Caspase-3 is considered the most important caspase executioner and is activated by any initiator caspases. Capase-3 activates DNA fragmentation factor, which in turn activates endonucleases to cleave nuclear DNA and ultimately leads to programmed cell death [67]. In the present study, we demonstrated that spinal cord caspase-3 activity significantly increased after compression injury, which was markedly alleviated by HSYA treatment. Although our findings did not explain all mechanisms underlying protective effects of HSYA, we postulated that alleviation of neuron apoptosis may be one of the key mechanisms.

Altogether, our study demonstrated that HSYA treatment can significantly attenuate secondary neuronal death through alleviated oxidative stress, inflammatory response, and neural apoptosis induced by primary SCI. Combining our results with multiple previous research which showed that HSYA can exert protective functions on neurons by inhibiting microglial activation [16] and aside from former indications and usages in cardiovascular and cerebral diseases, we further confirmed protective effect of HSYA and inferred its clinical utilization in preventing further damage after primary acute SCI. However, the present study poses several limitations. First, HSYA was only delivered via i.p. route. Future studies should further explore and elaborate differences in i.p. administration and oral route and how they affect bioavailability and metabolism of HSYA and potential active metabolites in vivo. Second, SCI model in SD rats may not fully represent spinal cord trauma in humans. Thus, effectiveness of HSYA treatment should be assessed in other models. Latest and more representative factors should be investigated in future studies to further support results of the present study.

## Conclusions

In conclusion, we provided first evidence showing that HSYA treatment significantly attenuates spinal cord edema and improves motor function outcomes in rats with clip-induced compression SCI model. The potential mechanism of this action is through ameliorating extent of oxidative stress and preventing release of pro-inflammatory molecules with inhibition of NF-κB. Reduction of apoptosis in the spinal cord may also contribute to protection of motor neuron after HSYA treatment. Although the exact protective mechanisms involved need to be further investigated, HSYA may be considered as potential effective therapeutic molecule for patients with spinal cord damage.

## Abbreviations

BBB: Basso, Beattie, and Bresnahan
BSCB: Blood–spinal cord barrier
CNS: Central nervous system
COX-2: Cyclooxygenase-2
ELISA: Enzyme-linked immunosorbent assay
EMSA: Electrophoretic mobility shift assay
HSYA: Hydroxysafflor yellow A
i.p.: Intraperitoneal injection
iNOS: Induced nitric oxide synthase
MDA: Malondialdehyde
MPO: Myeloperoxidase
NF-κB: Nuclear transcription factor-kappa B
NO: Nitric oxide
SCI: Spinal cord injury
SD: Sprague–Dawley
SOD: Superoxide dismutase
TBA: Trichloroacetic acid
